# Indications and Outcome in Patients Undergoing Left Atrial Appendage Closure—The Austrian LAAC Registry

**DOI:** 10.3390/jcm9103274

**Published:** 2020-10-13

**Authors:** David Zweiker, Raphael Sieghartsleitner, Lukas Fiedler, Gabor G. Toth, Olev Luha, Guenter Stix, Harald Gabriel, Paul Vock, Brigitte Lileg, Andreas Strouhal, Geort Delle-Karth, Michael Pfeffer, Josef Aichinger, Wolfgang Tkalec, Clemens Steinwender, Kurt Sihorsch, Ronald K. Binder, Martin Rammer, Fabian Barbieri, Silvana Mueller, Nicolas Verheyen, Klemens Ablasser, Andreas Zirlik, Daniel Scherr

**Affiliations:** 1 Department of Cardiology, Medical University of Graz, 8036 Graz, Austria; rsieghar@hotmail.com (R.S.); gabor.g.toth@medunigraz.at (G.G.T.); olev.luha@klinikum-graz.at (O.L.); nicolas.verheyen@medunigraz.at (N.V.); klemens.ablasser@medunigraz.at (K.A.); andreas.zirlik@medunigraz.at (A.Z.); daniel.scherr@medunigraz.at (D.S.); 2 Third Department for Cardiology and Intensive Care, Klinik Ottakring, 1160 Vienna, Austria; 3 Department of Internal Medicine, Cardiology and Nephrology, Hospital Wiener Neustadt, 2700 Wiener Neustadt, Austria; flukas@me.com (L.F.); michaelpfeffer@gmx.at (M.P.); 4 Department of Internal Medicine II, Medical University of Vienna, 1090 Vienna, Austria; guenter.stix@meduniwien.ac.at (G.S.); harald.gabriel@meduniwien.ac.at (H.G.); 5 Department of Internal Medicine III, University Hospital St. Pölten, 3100 St. Pölten, Austria; paul.vock@aon.at (P.V.); office@lileg-kardiologie.at (B.L.); 6 Department of Cardiology, Hospital Nord-Klinik Floridsdorf, 1210 Vienna, Austria; Andreas.Strouhal@gesundheitsverbund.at (A.S.); georg.delle-karth@gesundheitsverbund.at (G.D.-K.); 7 Department of Internal Medicine II, Elisabethinen Hospital, 4020 Linz, Austria; Josef.Aichinger@ordensklinikum.at (J.A.); wolfgang.tkalec@ordensklinikum.at (W.T.); 8 Department of Cardiology, Kepler University Hospital, 4020 Linz, Austria; hcsteinwender@hotmail.com (C.S.); kurt.sihorsch@kepleruniklinikum.at (K.S.); 9 Department of Internal Medicine II, Klinikum Wels-Grieskirchen, 4600 Wels, Austria; Ronald.Binder@klinikum-wegr.at (R.K.B.); martin.rammer@klinikum-wegr.at (M.R.); 10 Department of Internal Medicine III, Medical University of Innsbruck, 6020 Innsbruck, Austria; fabian.barbieri@i-med.ac.at (F.B.); silvana.mueller@tirol-kliniken.at (S.M.); 11 Department of Cardiology, Cardiovascular Research Institute Maastricht (CARIM), Maastricht University Medical Centre, 6229 ER Maastricht, The Netherlands

**Keywords:** atrial fibrillation, left atrial appendage, registry, stroke, bleeding

## Abstract

Background: Complete real-world data on the indications and outcomes of left atrial appendage closure (LAAC) outside of clinical trials are rare. In this study, we stratified patients undergoing LAAC by indication groups. Methods: This analysis of the national multicentre Austrian LAAC Registry comprised all patients that underwent LAAC up until 2018 at the currently active centres in Austria. The baseline characteristics, procedural details and outcomes between the following indication groups were compared: bleeding as an indication for LAAC (“bleeding” group) vs. thromboembolism despite oral anticoagulation (OAC; “thromboembolism” group) vs. an intolerance to OAC for reasons other than the above (“other” group). Results: The analysis included 186 patients, with 59.7% in the “bleeding” group, 8.1% in the “thromboembolism” group and 32.2% in the “other” group. The CHADS_2_ score was the highest in the “thromboembolism” group and the HAS-BLED score was the highest in the “bleeding” group. The procedural outcomes were similar between groups (implantation success, 97.3%), with major complications occurring in 7.0% of patients. One-year survival free from stroke, bleeding or LAAC-associated hospitalisation was 83.9%, 90.0% and 81.4% in the “bleeding”, “thromboembolism” and “other” groups, respectively (*p* = 0.891). Conclusions: In routine clinical practice, LAAC was used in a heterogeneous patient population with atrial fibrillation (AF) and contraindication, inefficacy or intolerance to OAC. The long-term outcome was favourable in all groups.

## 1. Introduction

Left atrial appendage closure (LAAC) has evolved as an alternative to oral anticoagulation (OAC) for stroke prevention in atrial fibrillation (AF) patients. This procedure is currently recommended in patients with AF, elevated stroke risk, and contraindications to long-term anticoagulation treatment (class IIb, level of evidence B) [1,2]. The European guidelines especially suggest that patients with a history of bleeding without reversible cause should be considered for LAAC [1]. However, in everyday clinical practice, patients with AF might have alternative indications as well, such as “intolerance” to OAC. This “intolerance” of OAC may range from a history of severe bleeding associated with OAC intake to personal patient preference. As a result, the treated patient population may be very heterogeneous regarding its comorbidities and risk profile. For example, the Evaluating Real-Life Clinical Outcomes in Atrial Fibrillation Patients Receiving the Watchman Left Atrial Appendage Closure Technology (EWOLUTION) registry reports a history of major bleeding only in the minority of patients [3]. As of today, there is only limited evidence regarding different indications for LAAC and their effects on procedural and long-term outcomes in daily clinical practice. The goal of the present study was therefore to evaluate the patient selection, outcomes and efficacy based on different indications of all patients that were included in the Austrian LAAC Registry until the end of 2017.

## 2. Materials and Methods

This study was an analysis of the prospective Austrian LAAC Registry (NCT03409159). This registry was initiated by the Austrian Society of Cardiology and comprises all LAAC procedures performed until the end of 2017 in all currently active centres in Austria. The ethics committee of the Medical University of Graz approved the study (29-355 ex 16/17). Information on the clinical follow up was completed using data from the Austrian government’s population registry.

### 2.1. Recruitment and Indications

The recruitment of patients for LAAC was left to the discretion of the treating physician and the participating centres. All centres were advised to select patients suitable for LAAC based on current guidelines [1]. For further analysis, patients were stratified by the primary indication leading to the decision to perform LAAC. All patients with a history of major bleeding were categorised into the bleeding group. The second group (thromboembolism) comprised all patients with a history of cerebral or peripheral thromboembolism, with or without OAC. All residual patients were aggregated in the other group. Patients with both bleeding and thromboembolism events were stratified according to the index event on which basis the decision to perform LAAC was made.

### 2.2. Procedure

The selection of the device was left to the operators’ or institutes’ discretion. All patients that were enrolled in the registry received either Watchman™ (Boston Scientific, Marlborough, MA, USA), Amplatzer Cardiac Plug™ (Abbott Laboratories, North Chicago, IL, USA) or Amplatzer Amulet™ (Abbott Laboratories) devices. Procedures were performed according to the vendors’ standard operating protocol.

### 2.3. Antithrombotic Treatment

Pre-, peri- and post-procedural antithrombotic regimens were chosen by the treating physicians after considering the manufacturers’ guidelines, the patients’ comorbidities and their individual risk profile.

### 2.4. Follow-Up

Patients were followed according to the respective protocols of the participating centres, including transoesophageal echocardiography (TOE) being performed 3–6 months after LAAC and further visits every 6 to 12 months to the implanting centre or peripheral institutions. The follow-up data were analysed until 31 December 2017.

### 2.5. Data Collection

The registry parameters were based on the European Heart Rhythm Association (EHRA)/European Association of Percutaneous Cardiovascular Interventions (EAPCI) consensus statement on LAAC [4]. The data collection was performed either by an external reviewer or by a local representative. All data were collected and analysed anonymously. In addition to the regular follow-ups, all available databases of hospital associations were searched for any readmissions at other hospitals. Mortality data were assessed via a search through the Austrian government’s population registry (POPREG, Statistics Austria) [5]. The date of the last follow-up was defined as either the last clinical visit or the last day of available survival data according to the population registry.

### 2.6. Endpoints

Complications were defined as follows: procedural major complication—any procedural complication requiring an invasive intervention, procedural minor complication—any other procedural complication, cardiac tamponade—pericardial effusion requiring pericardiocentesis or surgery, access site complication—any access site complication requiring intervention, ischemic stroke—clinically relevant ischemic stroke according to current guidelines, shock—hypotension requiring catecholamines, cardiopulmonary resuscitation—cardiac arrest requiring cardiopulmonary resuscitation, acute kidney injury—an increase of serum creatinine by ≥1.5 times from baseline within <7 days, bleeding—any bleeding requiring rehospitalisation, stroke or thromboembolism—any ischemic stroke or peripheral thromboembolism requiring hospitalisation, hospitalisation due to LAAC—any rehospitalisation that was either a direct consequence of the LAAC procedure or was caused by malfunction of the LAAC device.

We defined residual flow to the left atrial appendage as any colour Doppler flow >1 mm in diameter as detected using TOE. The residual flow was stratified into minor flow (>1 and ≤5 mm) and major flow (>5 mm).

### 2.7. Statistical Analysis

We used IBM SPSS 20 (IBM, Armonk, NY, USA) for data analysis. Values were expressed as count (proportion in percent), mean ± standard deviation or median (interquartile range), as appropriate. For bivariate analysis, we used ANOVA in normally distributed values (based on the Kolmogorov–Smirnov Z test); otherwise, the Kruskal–Wallis test was used. If a significant interaction between groups was observed, we performed post-hoc testing using the Kruskal–Wallis test and LSD. For categorical values, we used Fisher’s exact test. The *p*-values were adjusted according to the Bonferroni correction, if appropriate. Unless stated otherwise, distinct *p*-values in the text refer to between-group differences.

To predict the number of bleeding and stroke events based on individual CHA_2_DS_2_-VASc and HAS-BLED scores, we used the data of Olesen et al. [6,7], as outlined by LaHaye et al. [8]. Where applicable, we used the adjusted score instead of the reported score. After the calculation of the individual risk, we adjusted it according to the length of observation. All individual risks were added together to predict the number of bleeding and thromboembolism events per patient-year for the whole population, which was then compared to the observed stroke and thromboembolism event rate.

## 3. Results

**Figure 1:**
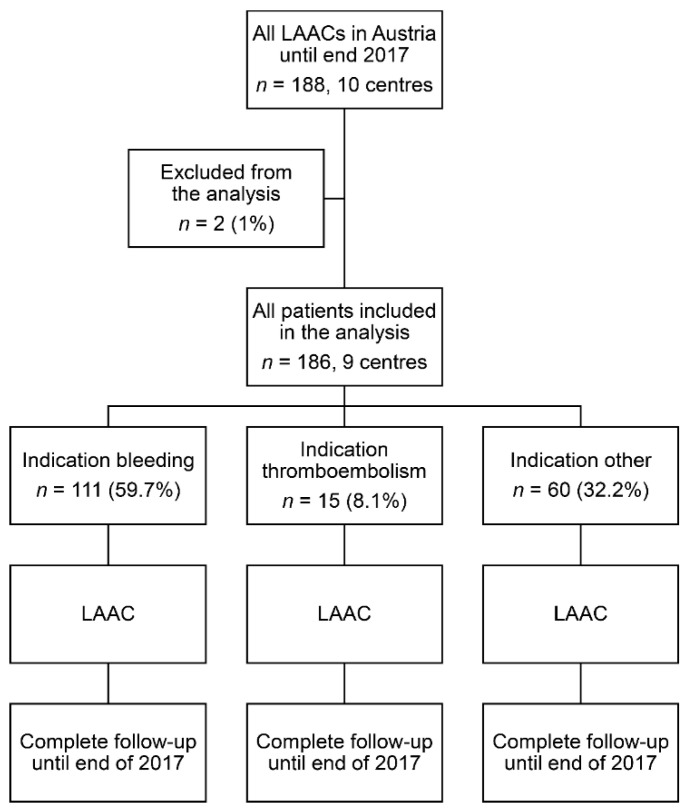
Study flowchart. LAAC: left atrial appendage closure.

Between November 2010 to December 2017, 186 consecutive patients undergoing LAAC at nine centres in Austria were included in this analysis (Figure 1). A median of 17 procedures was performed at each centre, with a range from 8 to 43 procedures. This corresponded to a median LAAC implantation rate of 7.6 per year and centre (range, 1.1 to 12.3). Two patients of a currently inactive centre had to be excluded because the procedural and follow-up data could not be provided by the implanting centre. The median age was 75 (interquartile range, 70–79) years and 37.6% were female. The baseline characteristics are shown in Table 1.

### 3.1. Indications

**Figure 2:**
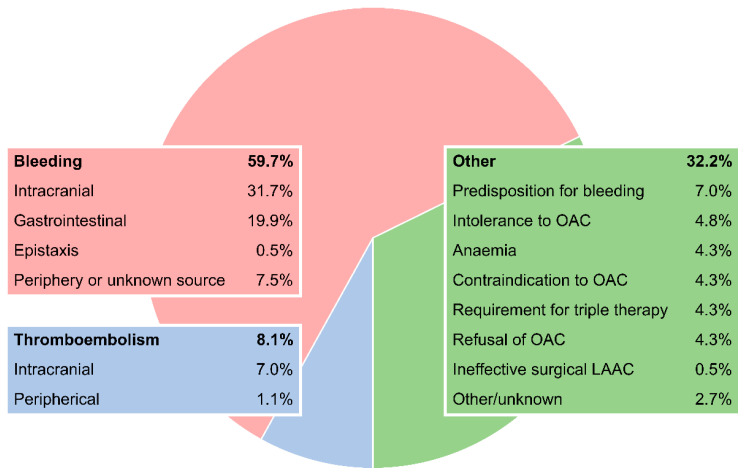
Indications of LAAC in the Austrian LAAC Registry. LAAC: left atrial appendage closure; OAC: oral anticoagulation.

A history of bleeding was present in 59.7% (*n* = 111) of patients (bleeding group), with most of the patients having suffered from intracranial bleeding (31.7%, Figure 2). The rate of intracerebral haemorrhage (23.1%) in this group exceeded other causes of intracranial bleeding (subdural hematoma: 4.8%, subarachnoid bleeding: 3.8%, epidural haemorrhage: 0.5%, including patients with multiple intracranial bleedings). Gastrointestinal bleeding had occurred in 19.9%, followed by epistaxis (0.5%) and other (7.0%). The minority of patients with a history of bleeding (42.3%) had suffered a bleeding event without being on OAC; others had received either direct oral anticoagulants (DOACs) or vitamin K antagonists (VKAs, 28.85% each).

A total of 8.1% of patients received LAAC because of a history of thromboembolism despite OAC treatment, with 7.0% having a history of stroke and 1.1% having a history of peripheral embolism (thromboembolism group).

The third indication group (the other group) was very heterogeneous and comprised 32.2% of patients. Seven percent of patients had a predisposition for bleeding, such as gastrointestinal malformation (2.2%), Morbus Osler (1.6%) or cerebral malformation (1.1%). Other indications were intolerance to an OAC due to side effects without major bleeding (4.8%), anaemia without obvious cause (4.3%) and contraindication to an OAC (4.3%; for example, both liver and renal insufficiency). Another 4.3% of patients had LAAC because they refused a lifelong OAC and 4.3% received LAAC prior to a planned PCI to avoid triple antithrombotic therapy. One patient received LAAC to complete a previously performed insufficient surgical left appendage exclusion.

### 3.2. Basic Risk Profile

The CHA_2_DS_2_-VASc score of the overall cohort was 4.5 ± 1.4, the CHADS_2_ score was 2.8 ± 1.2 and the HAS-BLED score was 3.3 ± 0.9 (Table 1). Differences in comorbidities led to significantly different risk profiles between the pre-specified groups.

As expected, the HAS-BLED score was significantly higher in the bleeding group compared to the thromboembolism (*p* = 0.001) and other groups (*p* < 0.001) due to a more prevalent history of bleeding (100.0% vs. thromboembolism 26.7% vs. other 45.0%, *p* < 0.001).

Patients with a history of thromboembolism had a significantly higher thromboembolic risk (according to CHADS_2_ score) than the other group patients (*p* < 0.001) due to a significantly higher prevalence of stroke (93.3% vs. 18.3%, *p* < 0.001).

Coronary artery disease was significantly less prevalent in thromboembolism patients (6.7%) compared to the other two groups (bleeding patients: 45.0%, *p* = 0.013; other patients: 50.0%, *p* = 0.008).

Patients in the other group had a significantly lower prevalence of transitory ischemic attack, stroke or thromboembolism (28.3%) than the patients of other groups (bleeding group patients 47.7%, thromboembolism group patients 86.7%). Consequently, the CHADS_2_ score and HAS-BLED score were lowest in those patients.

### 3.3. Antithrombotic Treatment before LAAC

Of the whole patient population, 42.0% had received oral anticoagulation (DOAC, 29.6%; VKA, 12.4%) before the LAAC procedure (Table S1). The others had received low molecular weight heparin (LMWH, 23.7%), aspirin (23.1%), P2Y_12_ inhibitors (mostly clopidogrel, 14.0%) or dual antiplatelet therapy (8.1%). DOAC therapy prior to LAAC was significantly more prevalent in the thromboembolism group (80.0%) compared to the remaining groups (bleeding group 23.4%, other group 28.3%, *p* < 0.01).

### 3.4. Procedure

The LAAC procedure was combined with other procedures in 14.3% of cases. The most frequent simultaneous procedure was the closure of a patent foramen ovale (10.7%), which was performed numerically more often in the thromboembolism group (26.7%) than in the bleeding group (7.1%) and the other group (12.7%, *p*_overall_ = 0.055, Table S2). Other procedures were transcatheter mitral-valve repair (1.8%) or coronary angiography and/or percutaneous coronary intervention (1.8%). Cases were almost equally shared between the Amplatzer Cardiac Plug™ or Amplatzer Amulet™ device (52.2%) and the Watchman™ device (46.8%). More details about the procedure and procedural outcome can be found in Table S2.

### 3.5. Procedural Outcome

The median diameter of the implanted devices was 25 mm (IQR 24–27). The LAAC device was implanted successfully during the first procedure in 97.3% of patients. The reasons for implantation failure were technical difficulties (*n* = 3, 1.6%), intraprocedurally detected LAA thrombus (*n* = 1, 0.5%) and dislocation due to LAA anatomy (*n* = 1, 0.5%). The procedure duration (without other concomitant procedures) ranged from 20 min to 3.5 h (median, 70 min). Medians of 15 minutes (interquartile range, 11–23) of fluoroscopy and 100 mL (66–148) of contrast were needed, without significant differences between the indication groups or between devices.

Major complications occurred in 7.0% of patients, with no significant differences between any of the groups. The most common complications were cardiac tamponade (3.2%), access site complication (2.2%), ischemic stroke (1.1%) and shock requiring catecholamines (1.1%). The procedural and in-hospital mortality was 0%.

When stratified by centre, periprocedural complications ranged from 0% to 25%. There was no correlation between the periprocedural complication rate and the implantation rate per year and centre (*ρ* = −0.201, *p* = 0.604).

### 3.6. Antithrombotic Treatment after LAAC

After the LAAC procedure, a majority of patients (52.7%) were prescribed dual antiplatelet therapy (mostly clopidogrel plus aspirin) for 1–6 months after LAAC (Table S3). Other patients received an OAC with a DOAC (14.0%), OAC with a VKA (5.9%), single antiplatelet therapy with aspirin (7.5%), single antiplatelet therapy with clopidogrel (5.4%) or no antithrombotic therapy at all (12.4%). Oral anticoagulants were prescribed significantly more often in the thromboembolism group than in the bleeding group (53.3% vs. 17.1%, *p* = 0.004). For OAC, phenprocoumon (17.1%), apixaban (46.4%), dabigatran (35.7%) or rivaroxaban (17.9%) were used.

After a median of 3 months (IQR 2–6), 43.5% of patients switched to a single antiplatelet drug regimen as a long-term therapy. In 39.2% of patients, no further antithrombotic therapy was prescribed. Only in the thromboembolism group, four patients (26.7% of all thromboembolism patients) switched to an OAC with a DOAC (20.0%) or a VKA (6.7%). The long-term antithrombotic therapy could not be determined in 15.1% of all patients.

### 3.7. Follow-Up

Data from follow-up TOEs, performed 96 ± 73 days after the LAAC procedure, were available for 59.7% of patients. A correct position of the LAAC device was documented in 98.9% of cases. However, in one patient, the LAAC device could not be detected in the LAA and was found in a pulmonary vein. In another patient, the position was deemed incorrect due to a large gap of 6 mm between the device and the LAA wall. A minor residual flow was found in 1.8% of patients and a thrombus attached to the device in 1.9%.

**Figure 3:**
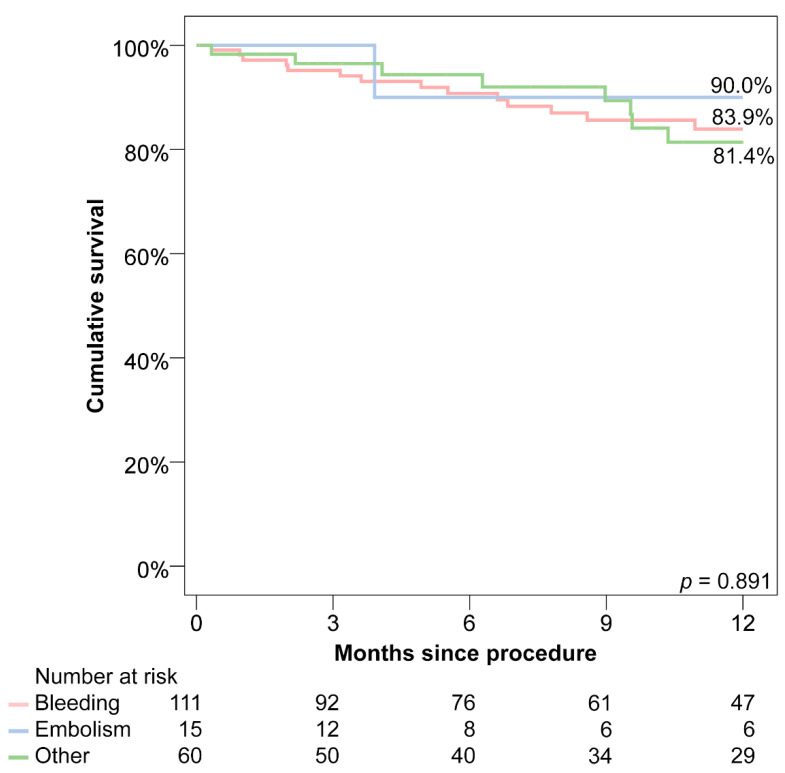
Kaplan-Maier curves for the combined endpoint of death, stroke, bleeding or LAAC-related rehospitalisation.

Long-term follow-up data were available for a mean of 477 ± 464 days, with 85.5% of patients reaching a follow-up after 90 days. In total, a combined 16.5% of the followed patients reached an endpoint of death, stroke, bleeding or LAAC-associated hospitalisation within the first year after the procedure, with no significant differences between indication groups (Table 2, Figure 3). During follow-ups, the all-cause mortality was 11.3%, while bleeding occurred in 7.0%, thromboembolism in 2.7%, ischaemic stroke in 1.6% and transient ischaemic attack in 0.5%. Patients that suffered a stroke or peripheral thromboembolism after LAAC were on single antiplatelet therapy (55.6%), no antithrombotic therapy (33.3%) or dual antiplatelet therapy (11.1%) during the event. Bleeding occurred in patients receiving single antiplatelet therapy (46.2%), dual antiplatelet therapy (30.8%) or no antithrombotic therapy (23.1%).

Three patients (1.6%) had to be readmitted to a hospital due to LAAC-device-related complications: One patient suffered from Dressler’s syndrome and one patient was admitted to the hospital for the administration of intravenous unfractionated heparin because of a large thrombus detected on the device using transthoracic echocardiography. In the third patient, a previously correctly implanted LAA occluder dislocated spontaneously and was found free-floating in a pulmonary vein. It had to be surgically removed. There was no significant difference between the indication groups in terms of the echocardiography results or long-term follow-up events.

### 3.8. Predicted vs. Observed Events

Based on the CHA_2_DS_2_-VASc score of our patient cohort, we predicted a yearly rate of stroke or peripheral thromboembolism of 8.6%. The predicted rate was the highest in the thromboembolism group (9.6%). Within the observation period of our study, we found an annual event rate of 3.7%, reflecting a significant relative reduction of 57% compared to the predicted rate (*p* = 0.035, Table 3).

According to the HAS-BLED score, our patient population had a predicted bleeding rate of 7.7% per year, with the highest predicted rate in the bleeding group patients (8.3%). However, we observed a yearly bleeding rate of 5.3%, leading to a relative risk reduction of 30%, which did not prove to be statistically significant (*p* = 0.454). The observed annual rate of both thromboembolic events and bleeding events was highest in the other group (embolic events, 4.5%; bleeding events, 5.7%) and lowest in the thromboembolism group (no events during follow-up).

## 4. Discussion

This analysis of the Austrian LAAC registry revealed three major findings: First, in clinical practice LAAC was performed in a heterogeneous patient population with a high risk of bleeding or thromboembolism and varying individual risk profiles. Second, LAAC was associated with a considerable risk of procedural complications, which were all managed successfully. Third, long-term thromboembolic and bleeding rates after LAAC were lower than expected compared to published historic controls.

### 4.1. Indications for LAAC

The current study highlights that all patients receiving LAAC in Austria had an elevated risk of thromboembolism and bleeding, as measured by validated scores. The mean CHA_2_DS_2_-VAsc score was 4.5 and the mean HAS-BLED score was 3.3 across all groups. Therefore, the medical or non-medical strategies used to prevent thromboembolic events were justified in these patients.

As LAAC is currently indicated as a second-line therapy, a specific index event or condition usually leads to the decision to perform LAAC. This especially applies in Austria because only a certain number of LAAC procedures each year are reimbursed. As a result, these “indications” for LAAC are well documented and LAAC can only be done for the highest risk population. In this study, the patients’ indications, which led to LAAC, were very heterogeneous. While bleeding (especially during OAC treatment) was the main reason in almost 60% of patients, in some patients, the predisposition for bleeding, refusal of an OAC or even the requirement for triple antithrombotic therapy for a limited duration were considered “contraindications” to OAC. Interestingly, 8.1% of patients received LAAC because they had experienced thromboembolic events, despite being on an adequate OAC.

There were significant differences in the baseline characteristics between indication groups, which could partly be explained by the indications themselves. However, there remained some other differences, such as a low incidence of coronary artery disease in the thromboembolism group, which endorses the importance of classifying patients into different indication groups.

Except for one study by Urena et al. [9] and the high-risk population of Hutt et al. [10], the thromboembolic risk in the Austrian LAAC registry, as predicted by the CHA_2_DS_2_-VASc score (median 5) or CHADS_2_ score (median 3), was higher than in previous studies, which report a median CHA_2_DS_2_-VASc score of 4 [3,11,12,13,14,15,16,17] or a median CHADS_2_ score of 2 [18,19]. The bleeding risk assessed by the HAS-BLED score was comparable to previous literature (median 3) [13,14,15,16,17].

To the authors’ knowledge, this is the first study to stratify patients by the indication to perform LAAC. Previous studies only report scarce data. In four multicentre registries, 72–93% had a history of bleeding [9,12,14,20]. In the large EWOLUTION registry, only 31% of patients had a history of haemorrhage [3], and Kefer et al. only mentioned having included patients with a previous embolism despite being on an OAC, without giving distinct numbers [13]. Patient choice was the main indication for LAAC in 27.1% of patients in the Left-Atrium-Appendage Occluder Register - GErmany (LAARGE) [20]. In the two existing randomised controlled trials, all AF patients with an elevated stroke risk were evaluated for LAAC and only patients without a contraindication to an OAC were included [11,18].

### 4.2. Procedural Outcome

In Austria, both Amplatzer™ and Watchman™ devices were used for LAAC almost equally (52.2% vs. 46.8%). Concomitant closure of the patient’s foramen ovale was performed most frequently in the thromboembolism group, probably to avoid a further thromboembolism via a right-to-left shunt in this group with a history of recurrent thromboembolism. While Berti reported a much lower rate of concomitant procedures in the national Italian registry (2.4%) [21], the rate of combined procedures in our analysis (14.3%) was similar to other studies [22,23]. The procedural duration was similar to previous reports [20,24,25].

With an incidence of 7.0%, major procedural complications occurred fairly often in this high-risk patient population. It is noteworthy that there were also two ischemic strokes during the LAAC device implantation. We did not notice any differences in major or other complications between the indication groups. Cardiac tamponade was the most frequent major complication. Fortunately, all complications were managed successfully until discharge and the mortality until discharge was 0%.

One reason for the comparatively high incidence of complications may have been the high-risk cohort undergoing LAAC. However, low operator experience may have also played a role, as the mean rate of procedures per year and centre was only 7.6. Interestingly, the centre with the lowest rate of yearly procedures (1.1) had the highest rate of periprocedural complications (25%). It is to be discussed whether patients would benefit from a centralisation of LAAC to a few centres to optimise the procedural results and post-procedural follow-up.

The implantation success rate of 97.3% was comparable to other studies, which included either Watchman [3,11,12,18,19] or Amplatzer [9,13,14,15,16,17] devices, reporting a success rate of 90.9 [18] to 99.1% [17]. Major adverse events in the literature were similar to this analysis and ranged from 2.2 [15] to 12.0% [13].

### 4.3. Post-Procedural Antithrombotic Treatment

We found that post-procedural antithrombotic treatment was tailored in accordance with the patients’ individual history. As a consequence, antithrombotic treatment after LAAC was very heterogeneous. For the first three months after the LAAC procedure, most of the patients received dual antiplatelet therapy. As a consequence of their medical history, the majority of patients in the thromboembolism group were on an OAC for 3–6 months after LAAC. However, despite their history of recurrent thromboembolism, even during OAC treatment, in almost 75% of thromboembolism patients, no long-term OAC was prescribed, but 67% either received a single antiplatelet medication or no further antithrombotic medication. Interestingly, 12.4% of patients received no antithrombotic therapy at all at the time of discharge. This approach contradicts current guidelines [4], which endorse a course of single antiplatelet therapy for at least two weeks, even in patients with very high bleeding risk. However, in this analysis, there was no signal showing markedly increased thromboembolism rates in these patients.

In the entire population, long-term medical therapy mostly consisted of single antiplatelet therapy (43.5%) or no therapy (39.2%).

In the majority of the existing analyses, dual antiplatelet therapy was recommended for 1–6 months [9,12,13,14,16,17], followed by aspirin for 3 months [14,16] or indefinitely [9,12,13]. Warfarin was used for the first 45 days in Watchman Left Atrial Appendage System for Embolic Protection in Patients with Atrial Fibrillation (PROTECT AF) [18] and Watchman LAA Closure Device in Patients With Atrial Fibrillation Versus Long Term Warfarin Therapy (PREVAIL) studies [11], and in 27% of patients in the EWOLUTION registry [3].

### 4.4. Long-Term Outcome

At the follow-up TOE, device-related complications, such as residual flow or thrombus formation, were rare (1.8% and 1.9%, respectively). Rehospitalisations due to the LAAC device itself were also rare (1.7%), and there was only one case of a dislocation of the LAA occluder. However, follow-up TOE data were missing for a considerable number of patients (40.3%), partly because the examination was performed in a department that was different from the implanting centre. This may have led to the under-reporting of long-term adverse events. Furthermore, regular follow-ups at the implanting centre may be beneficial after such a complicated procedure, as endorsed by guidelines [4].

During the first year of follow-up, 16.5% of patients experienced either death, stroke, peripheral thromboembolism, bleeding or an LAAC-associated hospitalisation. This fairly high long-term complication rate can be attributed to the high-risk patient population. When comparing the annual predicted thromboembolic and haemorrhagic events of historic controls to observed events in this study, a significant reduction of 57.0% of thromboembolic events was acknowledged, while the reduction of bleeding events (30.2%) was not significant. The reduction of events was most pronounced in the thromboembolism group, with no embolic or bleeding events during the whole follow-up period. This fact is of interest, as all of these patients had a history of recurrent embolic events before LAAC and only a quarter received an OAC as an indefinite therapy after the procedure. However, the small sample size of this group limits the validity of these results. The group with the highest bleeding and thromboembolic event rates, despite a moderate baseline risk profile, was the other group with thromboembolic events in 4.5% and bleeding events in 5.7%. This group also experienced the lowest reduction of events compared to historic controls. This fact might be explained by two reasons with unclear relevance: First, these patients could have more prevalent risk factors of bleeding or thromboembolism that are not addressed by traditional risk scores. Second, these patients might not profit as much from LAAC as other patients with a clear contraindication to an OAC. Further studies may be needed to further examine this heterogeneous subgroup. Due to the rather small sample size, only the reduction of annual embolic events of the whole population reached statistical significance (*p* = 0.035).

Because of the higher risk profile of our patient cohort, the thromboembolic rate of 3.7% was higher than in other studies, which reported events in 1.3 to 3.4% of patients [9,11,12,13,14,15,16,18,26]. The bleeding rate (5.3%) was also higher than existing analyses or registries, which reported rates of 0.0–4.8% [9,13,14,15,16,18,26], possibly because of the excessively high prevalence of previous bleeding in this cohort. The reduction of bleeding and embolic events compared to historic controls has been confirmed in other studies [12,26], which reported a bleeding rate reduction of 46% [26] and a reduction of thromboembolic events of 77–83% [12,26]. Concerning mortality, the results of this analysis (annual death rate = 8.6%) were similar to other studies (3.7 to 10.8%) [9,11,12,13,14,15,16,18,26].

### 4.5. Limitations

Large multi-centre real-world registries are valuable tools for monitoring the baseline factors and outcome of patients undergoing a procedure. We eliminated selection bias by including all patients undergoing LAAC in currently active centres in Austria. Due to external review at the majority of centres and the inclusion of data of the Austrian population registry, as well as searching the databases of hospital associations, we tried to minimise reporting bias and ensure a complete follow-up concerning mortality. However, the data quality cannot be compared to a controlled trial and a few patients had a follow-up of less than one year. In a proportion of patients, post-procedural TOE data was available because those patents were further managed at peripheral centres. Furthermore, the comparison with historic controls regarding the embolic and bleeding risk is always questionable, as risk scores never include all individual patient factors. This analysis only included Amplatzer™ and Watchman™ devices. The data may therefore not be extrapolated to other LAAC techniques, such as LAA suture ligation. Documentation of the procedural techniques was limited. Lastly, but importantly, the low sample size of the individual groups limited the validity of the results and may have led to an underestimation of factors that would have had a significant impact on outcome otherwise.

## 5. Conclusions

Patients undergoing LAAC in Austria have a high baseline risk of thromboembolic and bleeding events. While in a majority of patients, bleeding led to the decision to perform LAAC, other indications were heterogeneous, including thromboembolism, despite being on an OAC or patient preference. The LAAC procedure had a considerably high rate of short-term complications and a fairly elevated long-term complication rate. Long-term follow-ups suggested a reduction in thromboembolic events in the whole population when compared to historical controls.

## Figures and Tables

**Table 1:** Baseline characteristics of the treated patients, grouped by their indication for LAAC.

Parameter	Indication for LAAC			*p*-Value	
	Bleeding	Thromboembolism	Other	Overall	Post-Hoc
Number of patients	111	15	60	N/A	
Female	38.7%	20.0%	40.0%	0.355	
Age (years)	74 (70–78)	74 (62–76)	77 (72–81)	0.029	^§^
Body mass index, kg m^−2^	26 (24–30)	25 (22–30)	27 (24–31)	0.409	
CHA_2_DS_2_-VASc score	4.6 ± 1.4	4.8 ± 1.2	4.2 ± 1.5	0.150	
CHADS_2_ score	2.8 ± 1.2	3.8 ± 0.7	2.5 ± 1.2	<0.001	**^,‡‡^
HAS-BLED score	3.6 ± 0.7	2.9 ± 0.6	2.9 ± 0.9	<0.001	**^,^^†^^†^
Congestive heart failure	27.9%	6.7%	11.7%	0.017	^§^
Arterial hypertension	86.5%	86.7%	90.0%	0.780	
Diabetes mellitus	25.2%	40.0%	28.3%	0.462	
Transitory ischemic attack, stroke or thromboembolism	47.7%	100.0%	28.3%	<0.001	**^,^^†,^^‡‡^
Vascular disease	42.3%	20.0%	38.3%	0.270	
Uncontrolled hypertension	9.0%	0%	8.3%	0.762	
Abnormal renal function	15.3%	6.7%	13.3%	0.803	
Abnormal hepatic function	1.8%	0%	6.8%	0.240	
Stroke	40.5%	93.3%	18.3%	<0.001	**^,^^†^^,^^‡^^‡^
History of bleeding	100.0%	26.7%	45.0%	<0.001	**^,^^††^
Labile International Normalised Ratio values	2.7%	6.7%	1.7%	0.453	
Alcohol abuse	2.7%	0%	3.2%	0.655	
Coronary artery disease	45.0%	6.7%	50.0%	0.006	*^,^^‡^^‡^
Cerebral artery disease	15.0%	13.3%	10.9%	0.837	
Periphery artery disease	7.2%	13.3%	5.0%	0.389	
History of percutaneous intervention	22.5%	0%	31.7%	0.021	^‡^
History of coronary artery bypass grafting	13.5%	6.7%	5.0%	0.200	
Chronic obstructive pulmonary disease	18.2%	6.7%	7.3%	0.133	
Dialysis	1.0%	0%	0%	1.000	
Hyperlipoproteinemia	35.4%	64.3%	29.1%	0.059	
Paroxysmal AF	27.6%	26.7%	42.6%	0.165	

Paroxysmal atrial fibrillation (AF) was defined as a sinus rhythm detected on the ECG shortly before the procedure. The following symbols represent significant differences in the post-hoc testing (after a Bonferroni adjustment): bleeding vs. embolism: * *p* < 0.05, ** *p* < 0.01; bleeding vs. other: ^†^ *p* < 0.05, ^††^ *p* < 0.01; embolism vs. other: ^‡^ *p* < 0.05, ^‡‡^ *p* < 0.01; ^§^ no significant interaction in the post-hoc testing found.

**Table 2:** Follow up after LAAC.

Parameter	Indication for LAAC			*p*-Value
	Bleeding	Thromboembolism	Other	Overall
Follow-up duration (days)	474 ± 449	268 ± 203	535 ± 525	0.178
Combined endpoint (1 year death, stroke, bleeding or LAAC-associated hospitalisation)	16.1%	10.0%	18.6%	0.891
Death	9.0%	6.7%	16.7%	0.253
Bleeding	7.2%	0.0%	8.3%	0.668
Stroke, transient ischaemic attack or thromboembolism	4.5%	0.0%	6.7%	0.762
Ischemic stroke	1.8%	0.0%	1.7%	1.000
Transient ischaemic attack	0.9%	0.0%	0.0%	1.000
Thromboembolism	1.8%	0.0%	5.0%	0.572
Hospitalisation due to LAAC	0.9%	6.7%	1.7%	0.256
Any hospitalisation	28.8%	33.3%	30.0%	0.879

LAAC: left atrial appendage closure.

**Table 3:** Mean predicted annual stroke and bleeding events (as by CHA_2_DS_2_-VASc and HAS-BLED scores) compared to reported events in the Austrian LAAC Registry.

Parameter	All Patients	Indication for LAAC		
		Bleeding	Thromboembolism	Other
Annual embolic events				
Predicted	8.6%	8.9%	9.6%	7.8%
Observed	3.7%	3.5%	0.0%	4.5%
Relative reduction	−57.0%	−61.0%	−100.0%	−42.0%
*p*-Value	0.035 *	0.083	1.000	0.529
Annual bleeding events				
Predicted	7.7%	8.3%	6.6%	6.7%
Observed	5.3%	5.5%	0.0%	5.7%
Relative reduction	−30.2%	−33.2%	−100.0%	−15.6%
*p*-Value	0.454	0.483	1.000	1.000

LAAC: left atrial appendage closure. * *p* < 0.05

## Supplementary Materials

The following are available online at https://www.mdpi.com/2077-0383/9/10/3274/s1. Table S1: Preprocedural evaluation of treated patients, grouped by indication for LAAC. Table S2: Procedural outcome and follow-up echocardiography. Table S3: Post-procedural anticoagulation strategy. Table S4: Mean predicted annual stroke and bleeding events (as determined using CHA_2_DS_2_-VASc and HAS-BLED scores) compared to reported events in the Austrian LAAC Registry.
