# Supplementary material for: Indications and Outcome in Patients Undergoing Left Atrial Appendage Closure—The Austrian LAAC Registry
Source: J Clin Med. 2020 Oct 13;9(10):3274. doi: 10.3390/jcm9103274 (PMC7600032; doi:10.3390/jcm9103274)
Supplement: Supplementary file 1 [file jcm-09-03274-s001.pdf]

**Supplemental Table S1.** Preprocedural evaluation of treated patients, grouped by indication for LAAC.

| Parameter | Indication for LAAC | | | P value | |
|---|---|---|---|---|---|
| | Bleeding | Thromboembolism | Other | Overall | Post-hoc |
| **Laboratory** | | | | | |
| Haemoglobin, g/dL | 12.9±2.0 | 14.6±1.3 | 12.6±1.9 | 0.002 | **,‡‡ |
| ntProBNP, mg/dL | 979 (433-1913) | 618 (486-1464) | 755 (275-1528) | 0.534 | |
| Creatinine, mg/dL | 1.1 (1.0-1.4) | 1.1 (1.0-1.2) | 1.1 (1.0-2.3) | 0.339 | |
| **Echocardiography** | | | | | |
| Left-ventricular function | | | | 0.659 | |
| 35-50% | 16.2% | 6.7% | 13.3% | | |
| <35% | 13.5% | 12.7% | 6.7% | | |
| Patent foramen ovale | 9.9% | 20.0% | 11.7% | 0.510 | |
| LAA minimum orifice diameter, mm (n=58) | 17.0±4.3 | 19.5±2.7 | 16.1±3.7 | 0.277 | |
| LAA maximum orifice diameter, mm (n=58) | 20.8±4.6 | 23.0±3.2 | 19.3±4.1 | 0.222 | |
| **Baseline medication** | | | | | |
| VKA | 9.0% | 6.7% | 20.0% | 0.104 | |
| DOAC | 23.4% | 80.0% | 28.3% | <0.001 | **,‡‡ |
| Aspirin | 19.8% | 13.3% | 31.7% | 0.155 | |
| Adenosine diphosphate receptor inhibitor | 13.5% | 0% | 14.0% | 0.190 | |
| Low molecular weight heparin | 25.2% | 6.7% | 25.0% | 0.303 | |
| Dual antiplatelet therapy | 6.3% | 0% | 13.3% | 0.177 | |

DOAC: direct oral anticoagulant; LAA: left atrial appendage; LAAC: left atrial appendage closure; VKA: vitamin K antagonist. The following symbols represent significant differences in post-hoc testing (after Bonferroni adjustment): bleeding vs embolism: ** p<0.01; bleeding vs other: †† p<0.01; embolism vs other: ‡‡ p<0.01.

**Supplemental Table S2**. Procedural outcome and follow-up echocardiography.

| Parameter | Indication for LAAC: Bleeding | Thromboembolism | Other | P value: Overall | Post-hoc |
|---|---|---|---|---|---|
| **Technical details** | | | | | |
| LAAC only | 89.8% | 73.3% | 81.8% | 0.113 | |
| Concomitant PFO closure | 7.1% | 26.7% | 12.7% | 0.055 | |
| Concomitant transcatheter mitral-valve repair | 1.0% | 0.0% | 3.6% | 0.467 | |
| Concomitant coronary angiography +/- percutaneous intervention | 3.1% | 0.0% | 0.0% | 0.663 | |
| Implanted device | | | | 0.038 | § |
| Amplatzer™ | 47.7% | 86.7% | 51.7% | | |
| Watchman™ | 50.5% | 13.3% | 48.3% | | |
| other/none implanted | 1.8% | 0.0% | 0.0% | | |
| Size, mm | 25 (24-27) | 25 (22-28) | 25 (22-28) | 0.743 | |
| **Outcome** | | | | | |
| Primary implantation success | 97.3% | 100.0% | 96.7% | 1.000 | |
| Minor residual flow | 3.6% | 0.0% | 3.3% | 1.000 | |
| Procedure time, min (n=109) | 65 (51-89) | 60 (55-65) | 86 (60-110) | 0.088 | |
| Procedure time without concomitant procedures, min (n=73) | 64 (51-95) | 65 (55-182) | 85 (64-96) | 0.366 | |
| Dose area product, µGym² (n=117) | 4220 (1058-8901) | 8319 (6052-10428) | 6929 (1218-12485) | 0.149 | |
| Fluoroscopy time, min (n=123) | 15 (11-24) | 14 (11-32) | 17 (12-22) | 0.795 | |
| Amount of contrast, mL (n=148) | 96 (64-146) | 106 (70-145) | 106 (66-154) | 0.595 | |
| Hospitalization duration, days (n=168) | 3 (2-5) | 2 (2-6) | 3 (2-7) | 0.225 | |
| Procedural major complication | 4.5% | 13.3% | 10.0% | 0.159 | |
| Cardiac tamponade | 2.7% | 6.7% | 3.3% | 0.512 | |
| Access site complication | 1.8% | 6.7% | 1.7% | 0.376 | |
| Ischemic stroke | 0.0% | 0.0% | 3.3% | 0.258 | |
| Shock | 0.9% | 0.0% | 1.7% | 1.000 | |
| Cardiac arrest | 0.9% | 0.0% | 0.0% | 1.000 | |
| Procedural minor complication | 9.9% | 13.3% | 16.7% | 0.443 | |
| Unplanned admission to intensive care unit | 4.5% | 6.7% | 6.7% | 0.679 | |

| | | | | | |
|---|---|---|---|---|---|
| Bleeding requiring blood transfusion | 0.9% | 0.0% | 8.3% | 0.030 | § |
| Acute kidney injury | 0.9% | 0.0% | 3.3% | 0.443 | |
| Echocardiography at follow up | | | | | |
| Duration to TOE, days (n=111) | 96±58 | 69±27 | 103±99 | 0.487 | |
| Residual flow (n=110) | 1.5% | 0.0% | 2.8% | 1.000 | |
| Major | 0.0% | 0.0% | 0.0% | | |
| Minor | 1.5% | 0.0% | 2.8% | | |
| Thrombus (n=108) | 1.5% | 0.0% | 2.9% | 1.000 | |

LAAC: left atrial appendage closure; PFO: patent foramen ovale; TOE: transoesophageal echocardiography. § no significant interaction between groups has been found in post-hoc testing.

**Supplemental Table S3**. Post-procedural anticoagulation strategy.

| Parameter | Indication for LAAC: Bleeding | Thrombo-embolism | Other | P value: Overall | Post-hoc |
|---|---|---|---|---|---|
| Short-term antithrombotic therapy | | | | | |
| Duration, months (n=160) | 3 (2-6) | 3 (3-6) | 3 (3-6) | 0.309 | |
| DAPT | 47.7% | 46.7% | 63.3% | 0.131 | |
| Oral anticoagulation | 17.1% | 53.3% | 16.7% | 0.008 | *,‡ |
| DOAC alone | 12.6% | 20.0% | 8.3% | | |
| VKA alone | 4.5% | 0.0% | 1.7% | | |
| DOAC + SAPT | 0.0% | 20.0% | 1.7% | | |
| VKA + SAPT | 0.0% | 13.3% | 5.0% | | |
| SAPT | 18.9% | 0.0% | 11.7% | 0.114 | |
| Aspirin | 10.8% | 0.0% | 3.3% | | |
| Clopidogrel | 4.5% | 0.0% | 8.3% | | |
| Aspirin + LMWH | 2.7% | 0.0% | 0.0% | | |
| Clopidogrel + LMWH | 0.9% | 0.0% | 0.0% | | |
| No antithrombotic therapy | 16.2% | 0.0% | 8.3% | 0.112 | |
| Long-term antithrombotic therapy | | | | | |
| SAPT | 42.3% | 33.3% | 48.3% | 0.560 | |
| Aspirin | 36.0% | 26.7% | 45.0% | | |
| Clopidogrel | 6.3% | 6.7% | 3.3% | | |
| No antithrombotic therapy | 40.5% | 33.3% | 38.3% | 0.869 | |
| Oral anticoagulation | 0.0% | 26.7% | 0.0% | <0.001 | **,‡‡ |
| DOAC | 0.0% | 20.0% | 0.0% | | |
| VKA | 0.0% | 6.7% | 0.0% | | |
| Other / not documented | 17.1% | 6.7% | 13.3% | 0.592 | |

DAPT: double antiplatelet therapy, DOAC: direct oral anticoagulant, LMWH: low molecular-weight heparin, SAPT: single antiplatelet therapy, VKA: vitamin K antagonist. The following symbols represent significant differences in post-hoc testing (after Bonferroni adjustment): bleeding vs embolism: * p<0.05, ** p<0.01; bleeding vs other: † p<0.05, †† p<0.01; embolism vs other: ‡ p<0.05, ‡‡ p<0.01; § no significant interaction in post-hoc testing found.
